# Supplementary material for: Water-Soluble Single-Benzene Chromophores: Excited State Dynamics and Fluorescence Detection
Source: Molecules. 2022 Aug 27;27(17):5522. doi: 10.3390/molecules27175522 (PMC9457774; doi:10.3390/molecules27175522)
Supplement: Supplementary file 1 [file molecules-27-05522-s001.zip › molecules-1849582-supplementary.pdf]

Supporting Information

for

Water-Soluble Single-Benzene Chromophores: Excited State Dynamics and Fluorescence Detection

Yingge Fan [1], Jin Ma [1], Huijing Liu [2,]* and Taihong Liu [2,]*

[1] Department of Chemical Engineering, Shaanxi Institute of Technology, Xi'an 710300, China
[2] Key Laboratory of Applied Surface and Colloid Chemistry of Ministry of Education, School of Chemistry and Chemical Engineering, Shaanxi Normal University, Xi'an 710062, China
* Correspondence: hjliu@snnu.edu.cn (H.L.); liuth121@snnu.edu.cn (T.L.).

Contents:

1. Supplementary UV-vis absorption and emission figures……………………….S2

2. Supplementary transient absorption (TA) spectra and data……………………..S3

3. Additional sensing performances……………………………………………..…S5

4. Determination of the detection limits……………………………………………S8

5. Performance comparison of present probes with the reported works……………S9

6. NMR spectra of the compounds……………………………………………..….S10

7. HRMS spectra of the compounds………………………………..……….S12

1. Supplementary figures

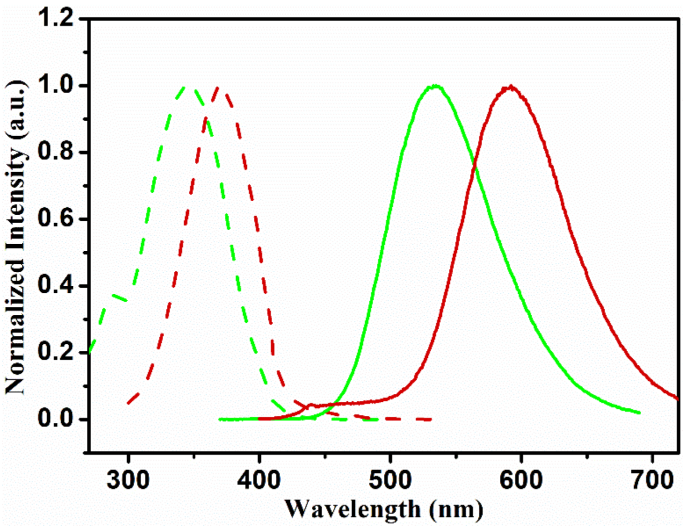

Figure S1. Normalized fluorescence excitation and emission spectra of **DAPA** (red lines) and **DAP-Na** (green lines) in water (c = 20.0 μM).

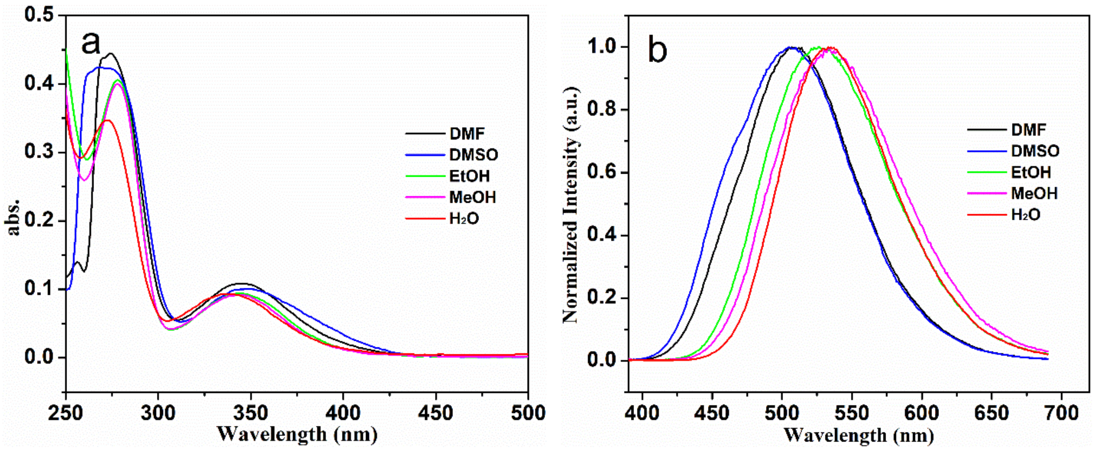

Figure S2. (a) UV-vis absorption and (b) normalized fluorescence emission spectra of **DAP-Na** in various solvents (λ_{ex} = 350 nm; c = 20.0 μM).

2. Supplementary transient absorption (TA) spectra and data

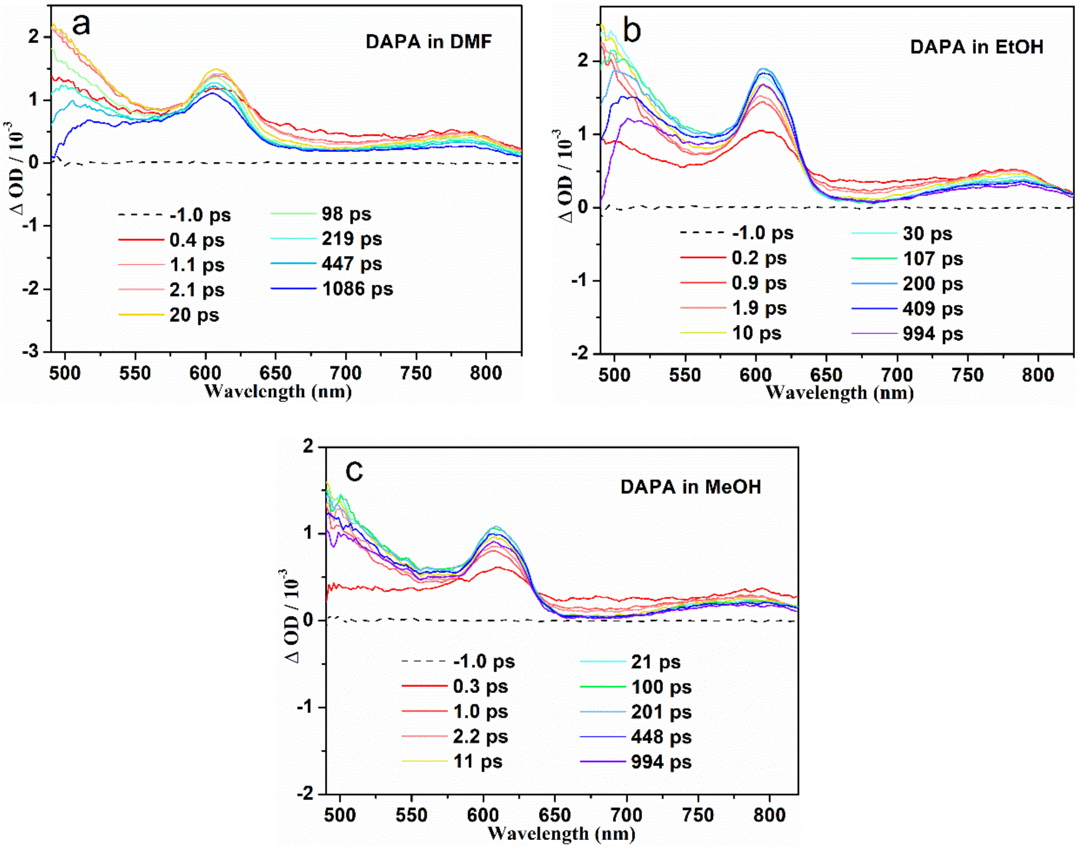

Figure S3. Femtosecond transient absorption spectra of **DAPA** in different solvents (DMF, ethanol and MeOH) upon excitation at fs-420 nm.

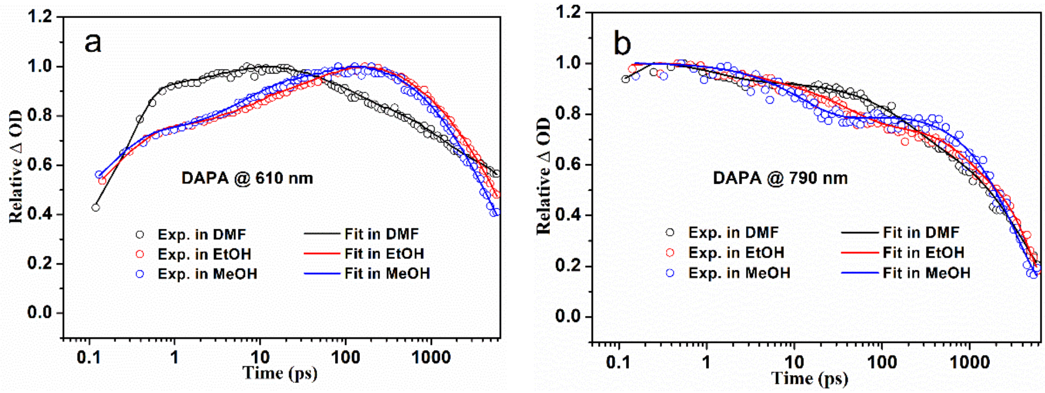

Figure S4. Experimental decay curves of **DAPA** in transient absorption and their fitting kinetic traces probed at 610 nm and 790 nm in three kinds of different solvents.

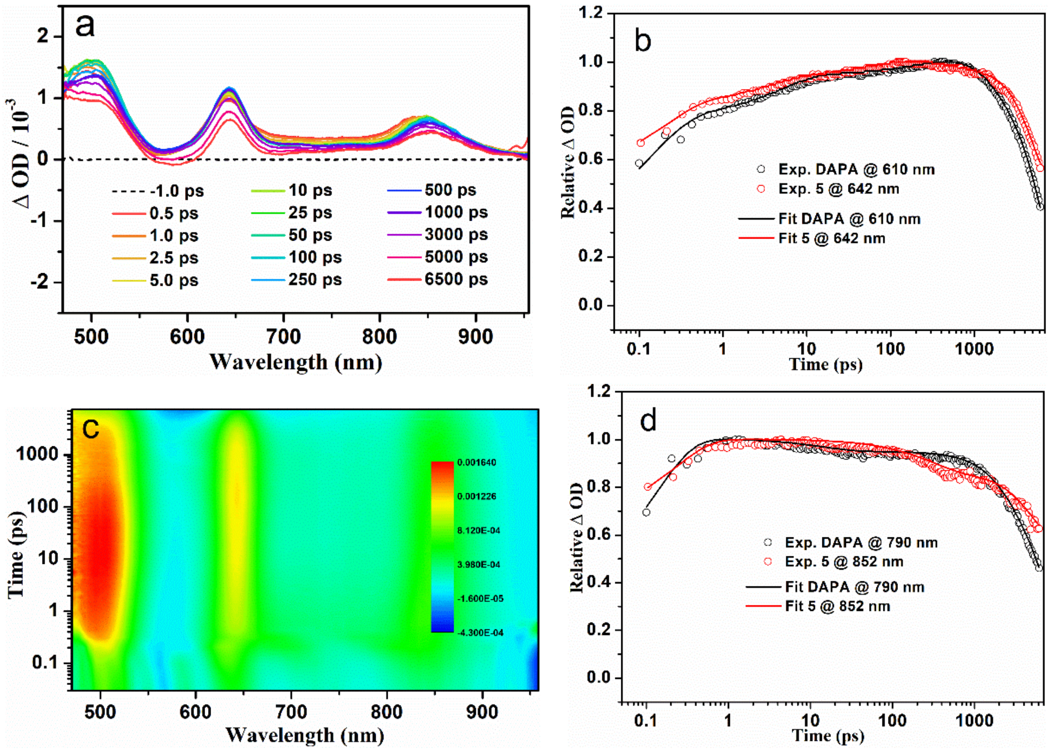

Figure S5. (a) Femtosecond transient absorption spectra of **5** upon excitation at fs-420 nm at various time delays in DMSO and (c) its species-associated spectra plots; (b) and (d) experimental decay curves in transient absorption of **DAPA** and **5** probed at different wavelengths and their fitting kinetic traces.

Table S1. Time constants of multiple exponential fitting of femtosecond TA data of different system, with relative amplitudes given for **DAPA** and **5** in different solvents.

Entry	Solvent	τ_1 (ps)	A1	τ_2 (ps)	A2	τ_3 (ns)	A3
**DAPA@610 nm**	DMF	4.6	-16%	75	35%	3.3	54%
	DMSO	4.7	-11%	212	12%	3.4	72%
	EtOH	6.4	-10%	56	9%	3.2	81%
	MeOH	5.4	7%	58	14%	3.6	79%
**DAPA@790 nm**	DMF	4.5	13%	167	22%	3.3	65%
	DMSO	7.9	-6%	145	26%	3.1	68%
	EtOH	5.7	12%	138	30%	3.1	58%
	MeOH	4.3	9%	115	17%	2.8	74%
**5@642 nm**	DMSO	3.1	7%	92	25%	2.6	68%
**5@852 nm**	DMSO	6.1	9%	107	31%	3.2	60%

3. Additional sensing performances

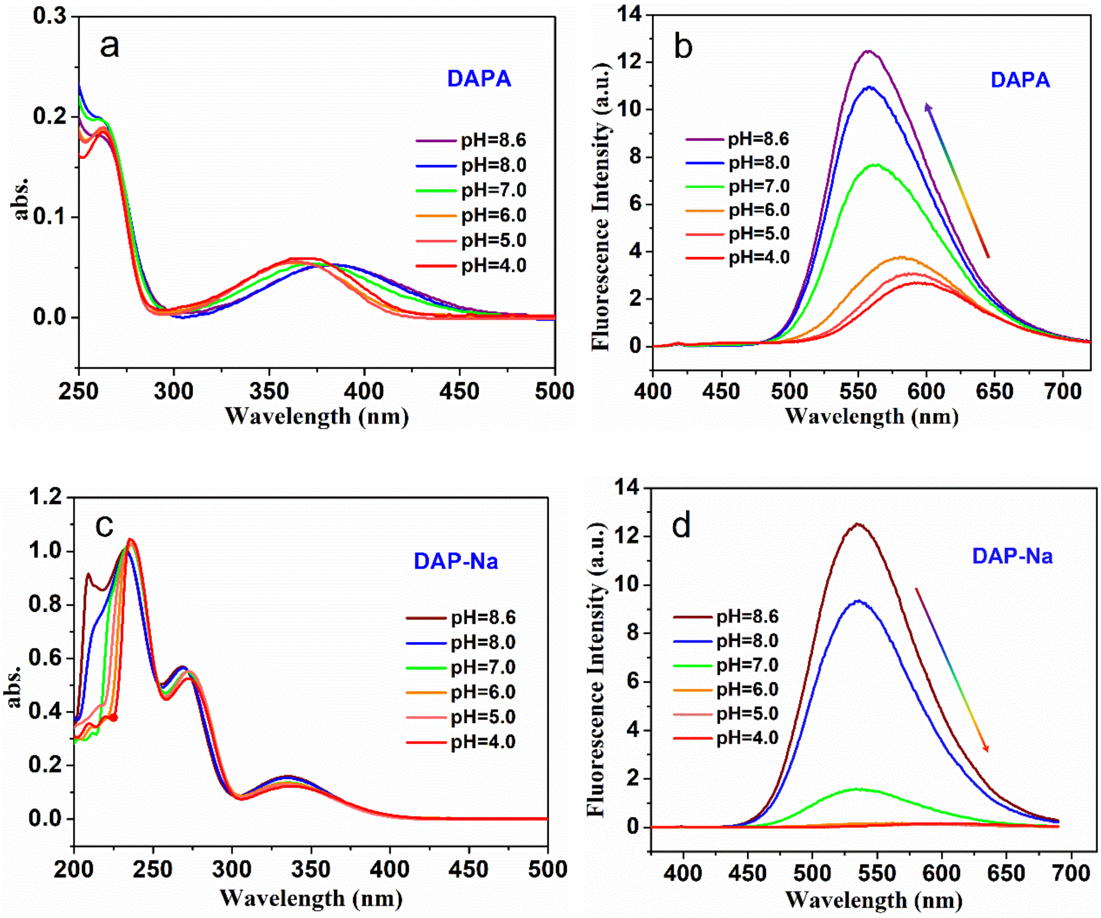

Figure S6. UV-vis absorption (a, c) and fluorescence emission spectra (b, d) of **DAPA** and **DAP-Na** in Britton-Robinson (B-R) buffer at different pH values (**DAPA**: λ_{ex} = 365 nm, **DAP-Na**: λ_{ex} = 350 nm, c = 20.0 μM).

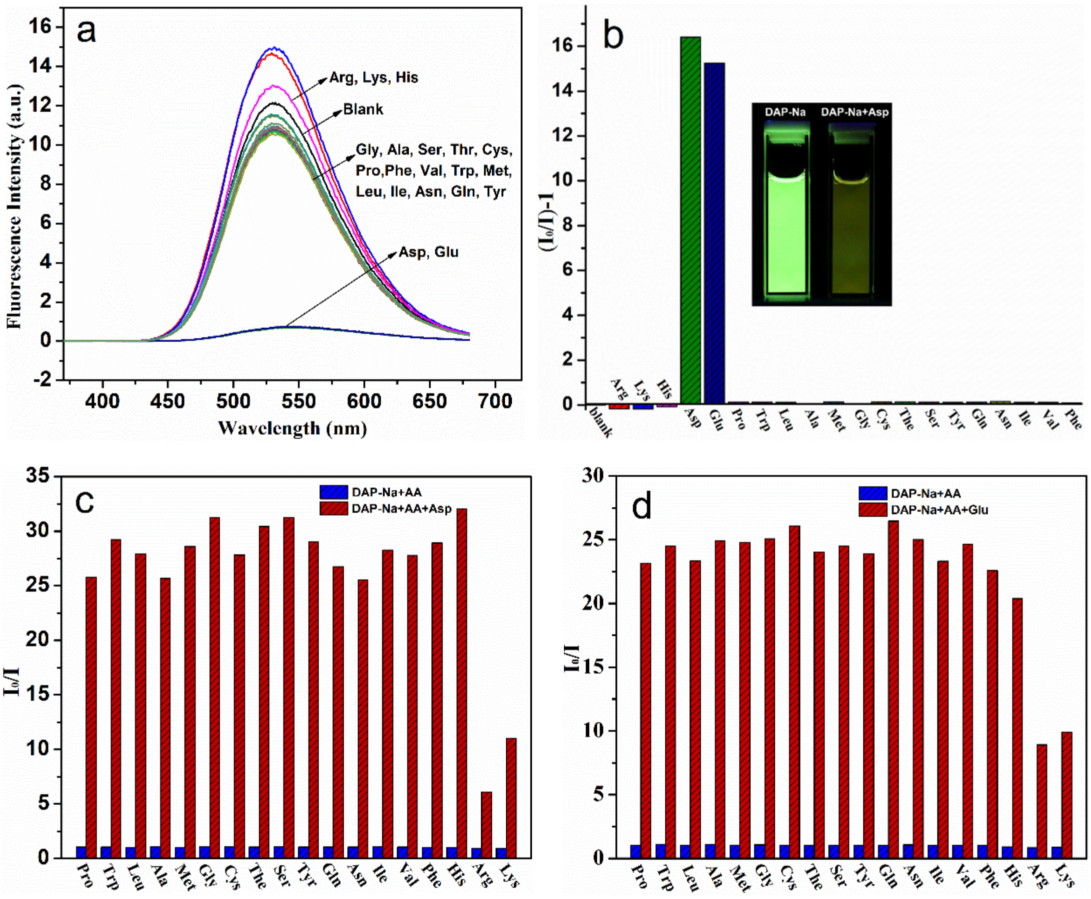

Figure S7. (a) Fluorescence emission spectra of **DAP-Na** in the absence and presence of various amino acids in water; (b) histogram style of fluorescence intensity changes, inset shows the photos of aqueous **DAP-Na** in the absence and presence of Asp under UV light (λ = 365 nm); anti-interference capabilities of **DAP-Na** to detect Asp (c) and Glu (d) in the presence of various amino acids (**DAP-Na**: c = 20 μM, amino acids: c = 100 μM).

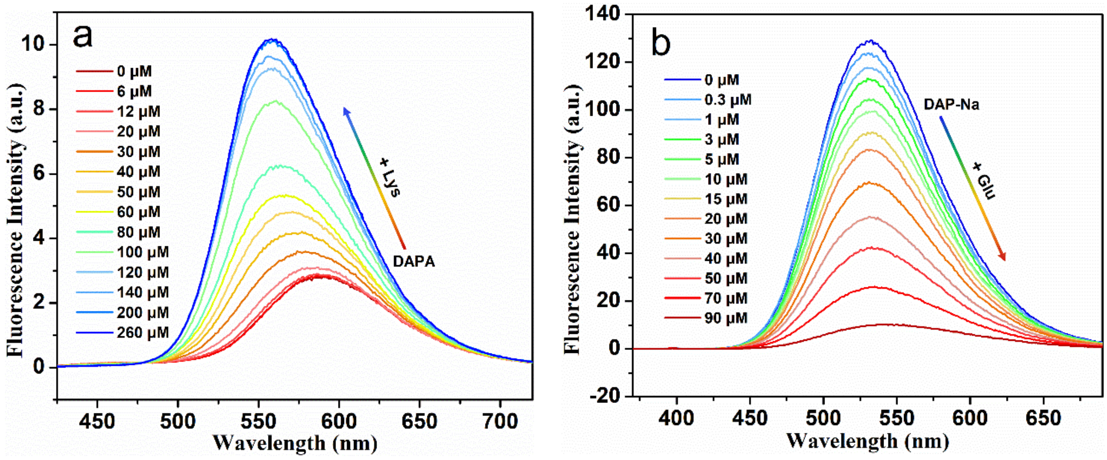

Figure S8. (a) Fluorescence emission spectra of **DAPA** upon titration of Lys (0 ~ 260 μM, pH = 7.05-9.13) in water; (b) fluorescence variation of **DAP-Na** upon titration of Glu (0 ~ 90 μM) in water (**DAPA**: λ_{ex} = 365 nm, **DAP-Na**: λ_{ex} = 350 nm, c = 20.0 μM).

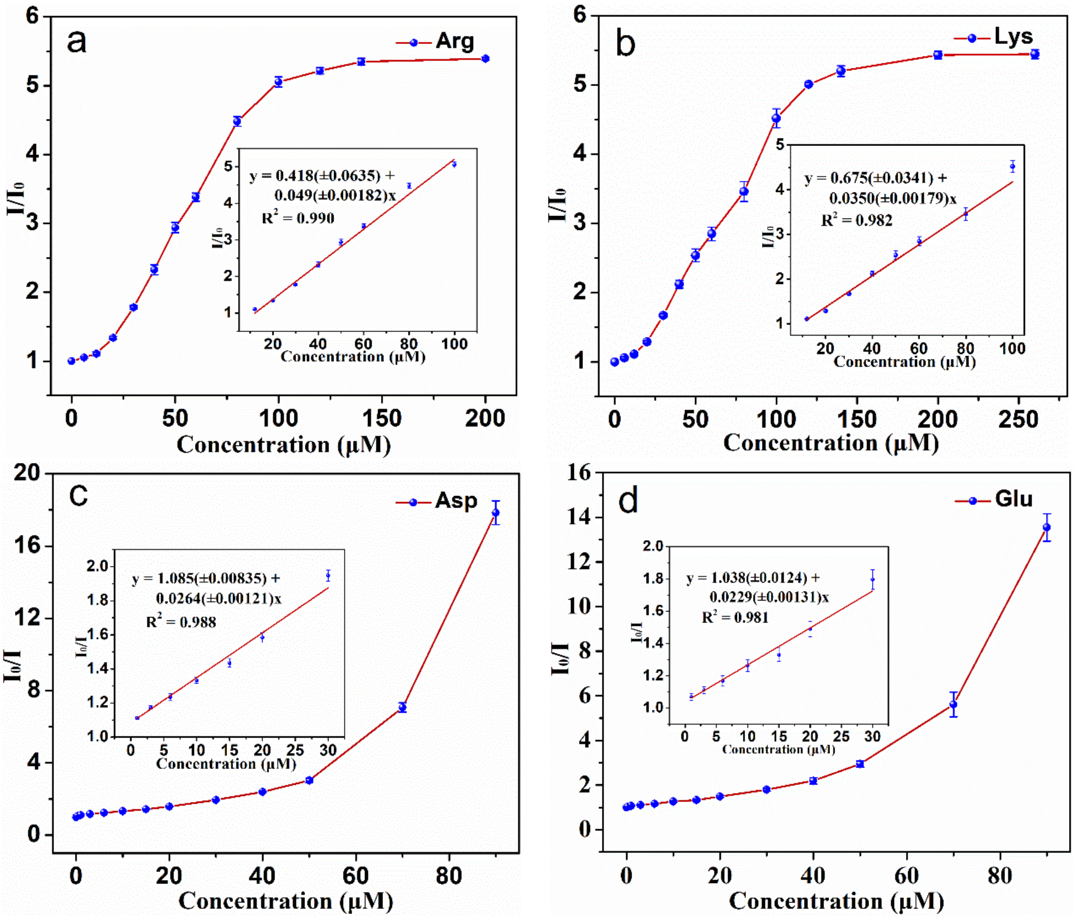

Figure S9. Fluorescence intensity ratio (I/I_0) at 558 nm of **DAPA** versus the concentration of Arg (a) and Lys (b) (*Inset*: linear relationship between I/I_0 and the concentration of Arg and Lys ranged from 12 to 100 μM); plots of fluorescence variation (I_0/I) at 531 nm of **DAP-Na** as a function of Asp (c) and Glu (d) concentration (*Inset*: linear relationship between I_0/I and the concentration of Asp and Glu ranged from 1 to 30 μM).

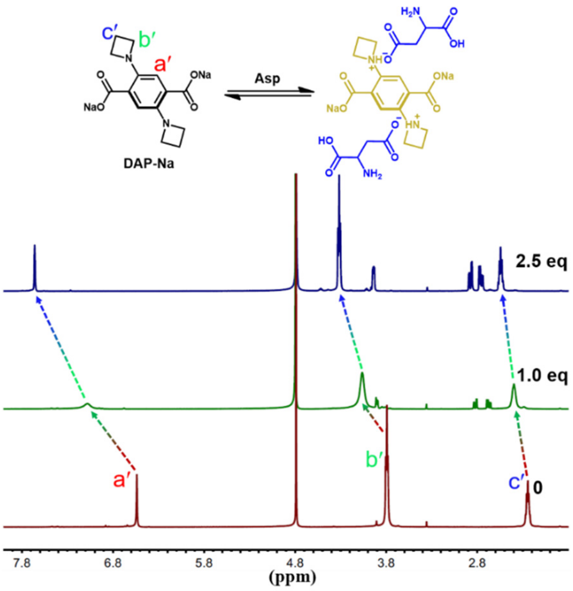

Figure S10. Partial ^1H NMR spectra of **DAP-Na** upon addition of Asp in D_2O.

4. Determination of the detection limits

Detection limits of **DAPA** and **DAP-Na** to basic amino acids (Arg and Lys) and acidic amino acids (Asp and Glu) were determined based on the fluorescence titration according to the following functions:

$$s_b = \sqrt{\frac{\sum_{i=1}^{n}(x_i - \bar{x})^2}{n-1}} \qquad (1)$$

$$S = \frac{\Delta I}{\Delta c} \qquad (2)$$

$$DL = \frac{3s_b}{S} \qquad (3)$$

Firstly, the standard deviation (S_b) was calculated by measuring the fluorescence intensity (x_i) of the probe as blank solution for 11 times and then got the average intensity ($\bar{x}$). By fitting the data into Function 1, the value of standard deviation was obtained. Secondly, a certain amount of analyte was added and the resulting variation of the fluorescence intensity (ΔI) was recorded. By fitting the data into Function 2, where ΔI is the variation of intensity, and Δc is the variation of analyte concentration, the value of precision S was calculated. Finally, the detection limit (DL) was calculated according to Function 3.

5. Performance comparison of present probes with the reported works

Table S2. Performance comparison of **DAPA** and **DAP-Na** for detection of different amino acids with the recently reported probes.

	Chromophore	Analyte	Approach	Solution Media	DL	Reference
No. 1	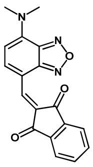	Arg/Lys	"turn-on" fluorescence/ colorimetry	DMF/H_2O (3/7) mixture	1.39 µM for Arg 1.10 µM for Lys	*ACS Appl. Bio Mater.* **2021**, *4*, 6558-6564
No. 2	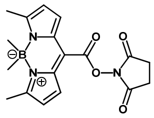	Arg/Lys	"turn-on" fluorescence/ colorimetry	PBS buffer (1% CH_3CN)	-- 0.062 µM for Lys	*J. Am. Chem. Soc.* **2020**, *142*, 9231-9239
No. 3	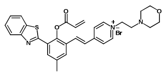	Arg	"turn-on" fluorescence	DMSO	2.24 µM for Arg	*Sens. Actuators B. Chem.* **2019**, *290*, 691-697
No. 4	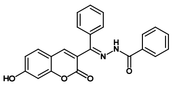	Arg/Lys	"turn-on" fluorescence/ colorimetry	CH_3CN/H_2O (9/1) mixture	--	*Sens. Actuators B. Chem.* **2019**, *279*, 400-409
No. 5	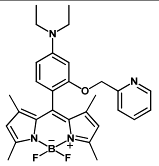	Asp/Glu	"turn-on" fluorescence/ colorimetry	DMSO/H_2O (3/97)	1 µM for Asp 5 µM for Glu	*Dyes Pigments* **2019**, *168*, 111-122
No. 6	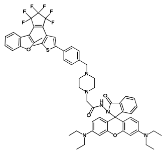	Arg	"turn-off" fluorescence/ colorimetry	CH_3CN/H_2O (4/1) mixture	2.2 µM for Arg	Sens. Actuators B **2017**, *247*, 26-35
No. 7	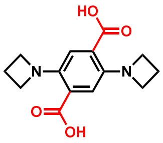	Arg/Lys	"turn-on" fluorescence	Aqueous	0.50 µM for Arg 0.41 µM for Lys	Present Work
No. 8	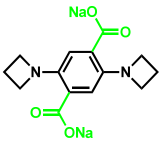	Asp/Glu	"turn-off" fluorescence	Aqueous	0.12 µM for Asp 0.16 µM for Glu	Present Work

6. NMR spectra of the compounds

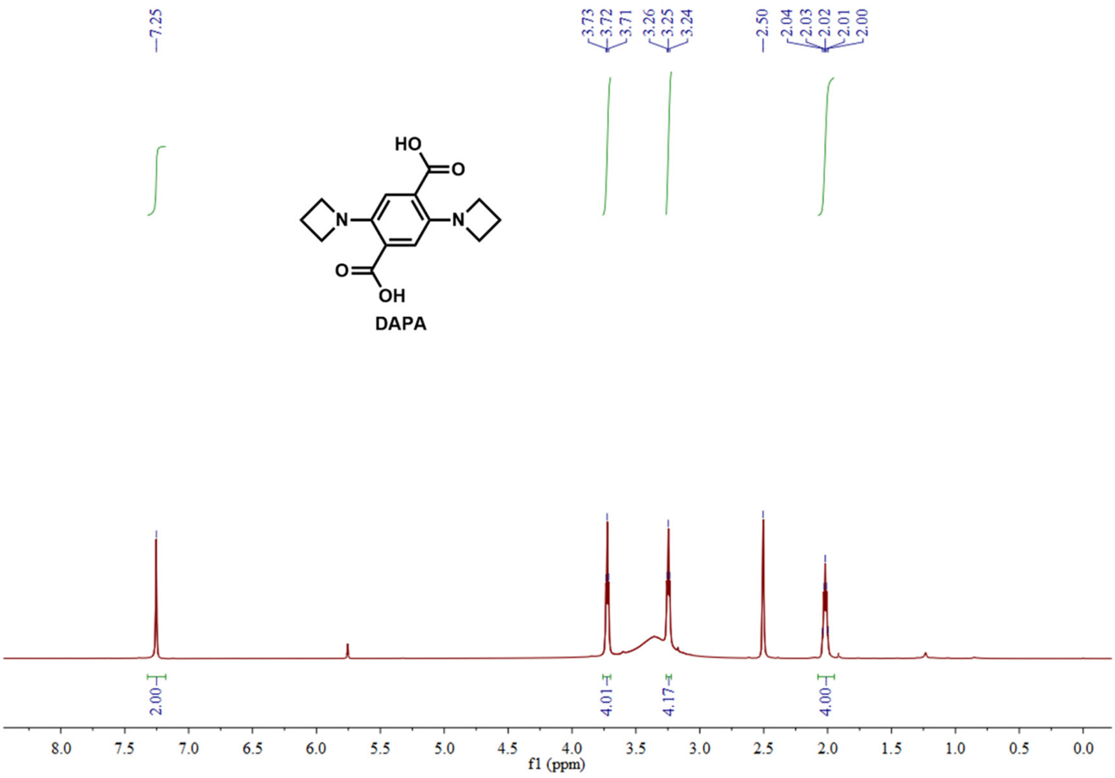

Figure S11. ^{1}H NMR spectrum of **DAPA** in d_6-DMSO.

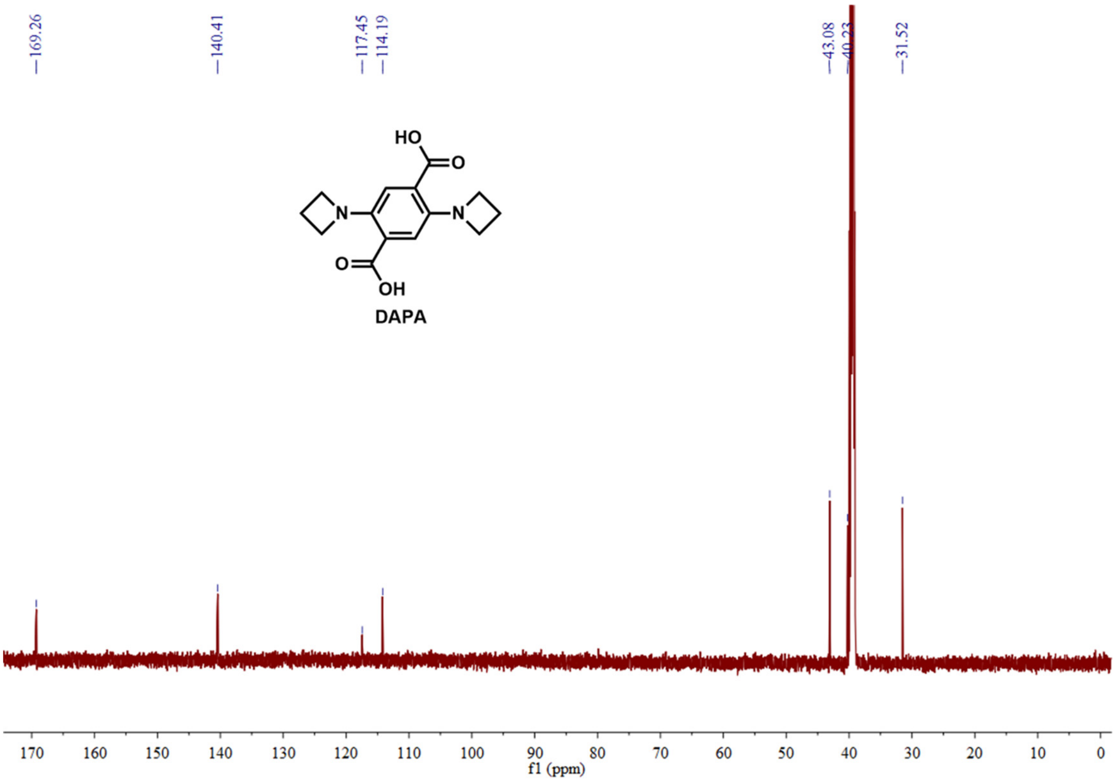

Figure S12. ^{13}C NMR spectrum of **DAPA** in d_6-DMSO.

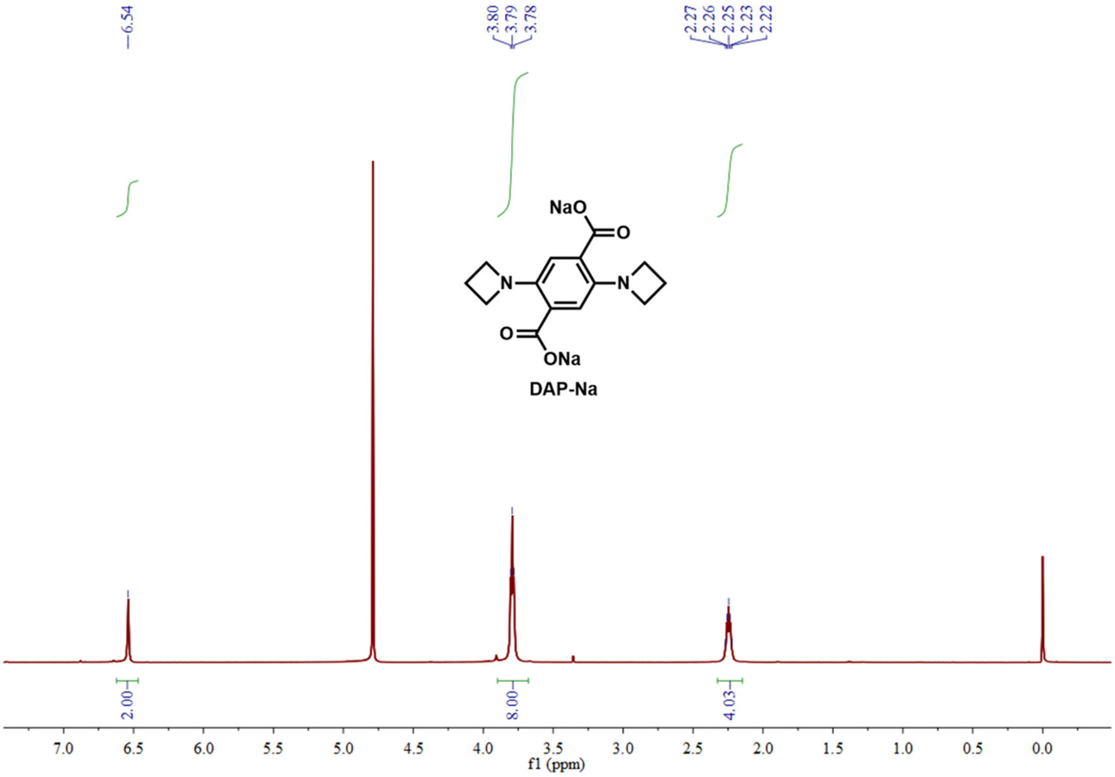

Figure S13. ^{1}H NMR spectrum of **DAP-Na** in D_2O.

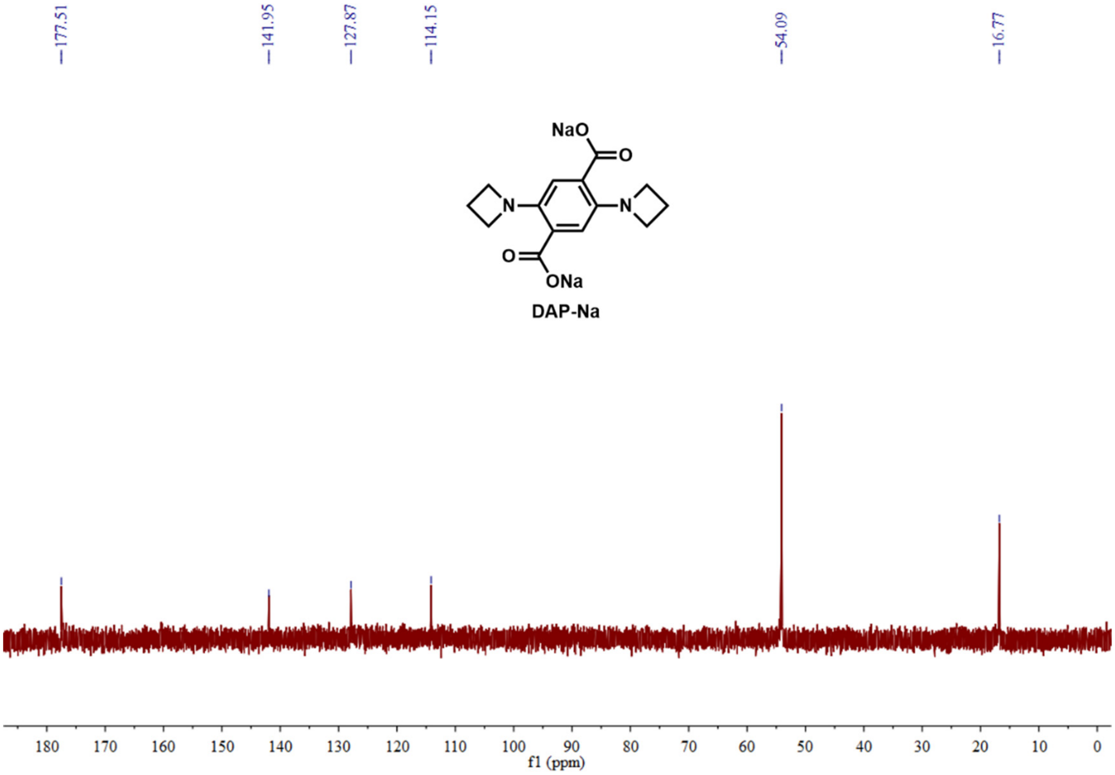

Figure S14. ^{13}C NMR spectrum of **DAP-Na** in D_2O.

7. HRMS spectra of the compounds

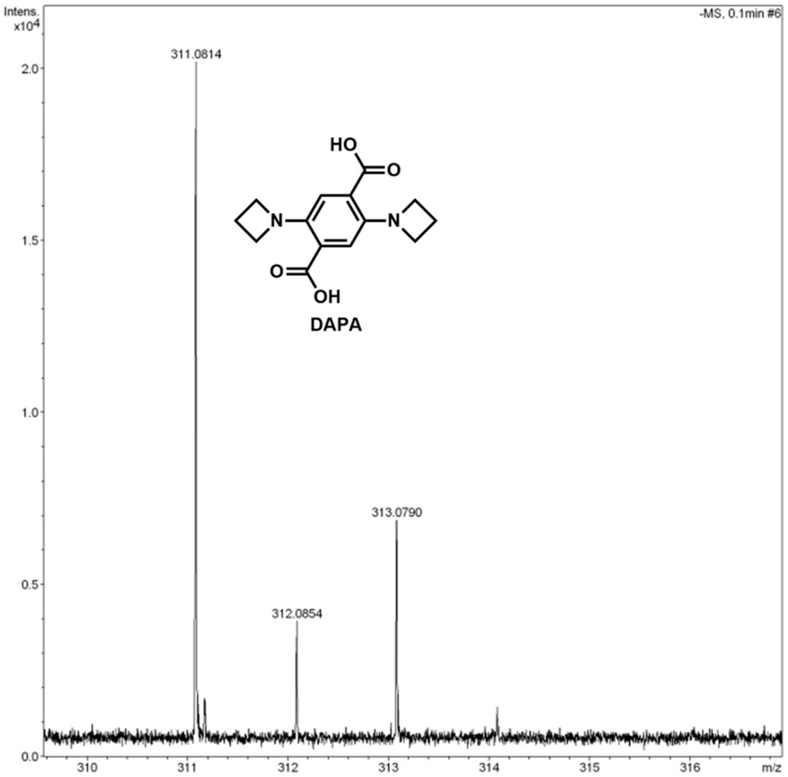

Figure S15. ESI-HRMS spectrum for **DAPA**.

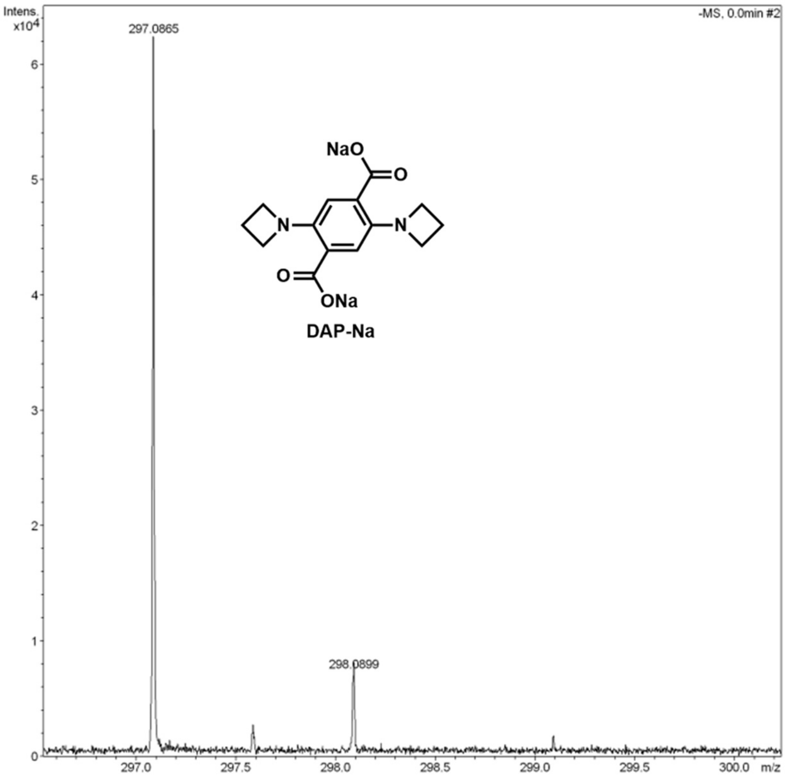

Figure S16. ESI-HRMS spectrum for **DAP-Na**.
